# Public health implementation of pathogen genomics: the role for accreditation and application of ISO standards

**DOI:** 10.1099/mgen.0.001097

**Published:** 2023-08-17

**Authors:** Susan A. Ballard, Norelle L. Sherry, Benjamin P. Howden

**Affiliations:** ^1^​ Microbiological Diagnostic Unit Public Health Laboratory, The University of Melbourne at the Peter Doherty Institute for Infection and Immunity, 792 Elizabeth St Melbourne, 3000, Australia; ^2^​ Department of Microbiology and Immunology, The University of Melbourne at the Peter Doherty Institute for Infection and Immunity, Melbourne, Australia; ^3^​ Department of Infectious Diseases, Austin Hospital, 145 Studley Rd., Heidelberg, Victoria 3084, Australia; ^4^​ Centre for Pathogen Genomics, The University of Melbourne, Melbourne, Australia

**Keywords:** whole-genome sequencing, international standards, laboratory accreditation

## Abstract

Pathogen genomics has transitioned rapidly from the research setting into a powerful tool now routinely used in public health microbiology, for surveillance, outbreak investigations and disease control. As these investigations can have significant public health, treatment and legal impacts, we must ensure the accuracy of these results through validation of testing processes. For laboratories working in this space, it is important to approach this work with a quality and accreditation framework in mind, working towards implementation of quality systems and test validation that meet international regulatory standards. Here we outline the key international standards and processes that lead toward accreditation for pathogen genomics.

## Pathogen genomics: from research to a routine public health tool

The emergence of pathogen genomics as a disruptive technology has led to major changes in clinical and public health microbiology, particularly over the last decade, as the technology has moved from use primarily in research settings, towards routine use in public health [1]. Genomic surveillance and outbreak investigations of pathogens of public health importance, such as foodborne pathogens, have been revolutionized, offering superior resolution, depth and breadth of data from a single laboratory test compared to previous methods [1, 2] (Table 1).

**Table 1.** Exemplar applications of pathogen genomics with significant ramifications

Organism	Application	Outcome and impact
* Vibrio cholerae *	Evolutionary history and transmission dynamics of the seventh known cholera pandemic in progress since the 1960s [36–38]	Identification of a point source introduction of *V. cholerae* from Nepal into Haiti followed by clonal expansion attributed to the UN peacekeeping base. Formal apology by UN, multimillion dollar assistance package to Haiti for improved access to healthcare, and addressing sanitation and water issues [39]
* Listeria monocytogenes *	Investigation of a prolonged multi-country outbreak of *L. monocytogenes* associated with contaminated frozen vegetables [40]	Identification of an outbreak of listeriosis (2015–2018), including 47 cases and 9 deaths across 5 countries (Denmark, Austria, Finland, Sweden and the UK). Epidemiological association with frozen corn implicated a freezing plant in Hungary as the source. Led to changes in *Listeria* sampling strategies, updated hygiene guidance and industry standards for frozen vegetables and communication of health risks. Importantly, it triggered a request from the European Commission for the integration of whole-genome sequencing (WGS) data in a One Health WGS system [40]
Severe acute respiratory syndrome coronavirus 2 (SARS-CoV-2)	Global emergence of the coronavirus disease 2019 (COVID-19) pandemic and monitoring of pathogen evolution and geographical spread [9, 41, 42]	Multiple international collaborative investigations on the origin of SARS-CoV-2 [43]. Divisive debate impacts on pandemic diplomacy [44]. Identification of a point source outbreak of SARS-CoV-2 directly linked to breaches in the Victorian Government Hotel Quarantine Program (see Box 1)
* Mycobacterium chimaera *	The origin of nosocomial outbreaks of post-operative *M. chimaera* [45, 46]	Identification of a point source origin of *M. chimaera* outbreak due to contamination at the heater–cooler unit devices (HCDs) manufacturing site [46]. Changed practice for positioning of HCDs, environmental monitoring and cleaning [47, 48]
		
* Mycobacterium tuberculosis *	Transmission dynamics and elucidation of antimicrobial resistance in *M. tuberculosis* [49–52]	Opportunities to implement precision medicine approaches for treatment of tuberculosis (TB) [52]

Box 1. **Case study on legal impact of pathogen genomics**

**Background**

In 2020, as the coronavirus disease 2019 (COVID-19) pandemic emerged, the primary measures of test, trace and contain were applied variously to prevent the spread of severe acute respiratory syndrome coronavirus 2 (SARS-CoV-2) around the world. In Australia this manifested in border closures, city- or state-wide lockdowns and supervised hotel quarantine programmes, along with significant investment in molecular testing to detect SARS-CoV-2 infections and the application of pathogen genomics to link positive cases to sources of infection. In the state of Victoria, Australia, real-time integration of genomic surveillance data was incorporated into the public health response to COVID-19 for the purpose of identification and elimination of spread of SARS-CoV-2. During the period 2020–2021, all COVID-positive samples were sequenced and phylogenetic results analysed in the context of epidemiological metadata. Results were used to identify sources of infection and prompted initiation of public health restrictions.

**Findings**

Between June and October 2020, a second COVID-19 wave occurred in Victoria, resulting in more than 18 000 infections, 800 deaths and a lockdown that lasted 112 days [9, 10]. Using WGS, approximately 99 % of infections could be traced back to infections within two security guards involved in Victoria’s hotel quarantine programme.

**Outcome**

The outcome of the genomics investigation ultimately led to a judicial enquiry to examine the policies, protocols and procedures in place with the hotel quarantine programme, along with decisions and actions of government agencies and contractors [10]. The report from the enquiry led to a major structural overhaul of the hotel quarantine programme, and the management of the state government’s COVID-19 response and ultimately led to the Department of Health facing charges of breaching the Occupational Health and Safety Act in September 2021 [53].

However, the implementation of genomics in public health has not always been straightforward, as genomics provides greater resolution to existing gold standard methods. With the rapid evolution and complexity of genomic workflows, and a paucity of international standards and quality assurance programmes, it is has been challenging to establish the veracity of genomics results. In essence, as we deploy genomics as a public health and clinical microbiology tool, how do we know that our results are ‘correct’? This is where international standards, and processes for test validation and laboratory accreditation, are critical, to ensure that reported results are credible and trustworthy.

Here, we share our experiences in implementing pathogen genomics in a public health laboratory setting and reflect on the role of accreditation in the deployment of the technology more broadly. The Microbiological Diagnostic Unit Public Health Laboratory (MDU PHL) is funded by the state department of health to perform surveillance, typing and reference testing on notifiable pathogens (including foodborne diseases, sexually transmitted infections, waterborne pathogens, invasive bacterial pathogens and antimicrobial-resistant pathogens). The laboratory has received specific funding to implement pathogen genomics in public health practice since 2015 and has generated over 11 000 bacterial sequences per year since 2017 [excluding >160 000 severe acute respiratory syndrome coronavirus 2 (SARS-CoV-2) sequences].

Early application of genomics for investigation of foodborne and antimicrobial-resistant outbreaks demonstrated the utility of the technology in the local context, supporting greater investment and a decision to transition the laboratory into a genomics-enabled public health laboratory [3–5]. As decisions on public health interventions started to be made based on our genomic results, a key question was – how do we ensure that our methods, interpretation and reporting are fit for purpose? The same questions were also relevant in the clinical realm, where genomics results could influence patient treatment (e.g. in multidrug-resistant infections), beyond academic interest.

## What is at stake? The importance of getting it ‘right’

The primary remit of public health laboratories (PHLs) is to provide microbiological data to support the actions and decision-making of public health units and other stakeholders. The portfolio of PHL activities may include surveillance, outbreak investigations (cases, food and environment), and forensic investigations. For example, genomics is being used in the food industry to characterize pathogens used in food production or existing as contaminants in ingredients and the manufacturing environment to understand their origin and develop processes to reduce risk to human health [6, 7].

The validation of new technologies such as those employed in genomic sequencing is essential to ensure that these results can be acted upon by public health teams, including legal proceedings (Table 1). For example, pathogen genomics data were critical in identifying the source of a large clonal wave of SARS-CoV-2 in Victoria in 2020; these data were examined critically during a public judicial enquiry, requiring the laboratory to demonstrate the validation and accreditation behind these data to attest to their credibility (Box 1). Other situations where genomics data may be used as legal evidence include accidental or purposeful release of a pathogen (including biothreat agents), or legal action against companies involved in food production or distribution (implicated in foodborne outbreaks) [8]. In some cases, genomics data inform responses that may lead to restriction on the activities of individuals or communities, testing the limits of patient privacy and confidentiality [9–11]. Equally important is the use of genomics data to inform the medical management of individual patients; in these cases, it is critical to demonstrate that the accuracy of these results to ensure patients and clinicians can confidently use these data to influence medical decision-making [12].

The introduction of large genomic surveillance platforms such as GenomeTrakr [13], AusTrakka [14], IRIDA [15] and INNUENDO [16] now has the potential to drive the detection of outbreaks prior to the emergence of epidemiological signals. However, this places a greater burden of proof on the scientific evidence to justify public health responses, and emphasizes the importance of standardization and validation to ensure that these results are scientifically correct and can be validly compared across borders. Any laboratories or platforms reporting on genomic data in a clinical or public health context should conform to best practices for bioinformatic analysis of genomic data, including implementation of data standards, maintenance of software, demonstration of data integrity and version traceability. This is critical for analyses that can have legal and medical consequences so that there is a complete audit trail regarding when, how and where evidence was generated.

## ISO standards and accreditation as a framework to ensure quality and consistency

International standards, developed and published by the International Organization for Standardization (ISO; https://www.iso.org/home.html), describe the best way to do something, and have been developed across a broad range of activities from describing best practice in delivering a service to making a product. They aim to assist with harmonization of processes and quality in testing across the globe, leading to acceptance of results, information, products and processes between countries. There are four key standards relevant to medical and biological testing laboratories, and these also apply to next-generation sequencing (NGS) technologies as they are embedded in public health and clinical contexts (Table 2). Two key standards apply to testing within laboratories: (i) ISO 17025, which ensures that laboratories can generate reproducible valid test results, allowing for the confident exchange of information between laboratories and other bodies, and (ii) ISO 15189, which applies specifically to medical laboratories.

**Table 2.** ISO standards applicable for laboratories performing NGS*

Standard	Name	Scope
ISO 15189 : 2022	Medical laboratories – requirements for quality and competence	Specifies requirements for quality and competence in medical laboratories. Includes development of quality management systems and auditing (internal and external) to assess competence
ISO 17025 : 2017	General requirements for the competence of testing and calibration laboratories	General requirements for competent and consistent operation of laboratories, ensuring they can deliver valid results. Includes risk management, how the laboratory deals with external bodies and harmonization of standards and processes
ISO 23418 : 2022	Microbiology of the food chain — whole-genome sequencing for typing and genomic characterization of bacteria — general requirements and guidance	Specifies minimum requirements for generation and analysis of whole-genome sequencing data of bacteria from the food chain

*ISO 20397-1 : 2021 and ISO 20397-2 : 2021, Massively Parallel Sequencing (MPS) parts 1 and 2, respectively, may also be of interest. These ISO standards specify guidelines for sequence library generation, quality assessments, post-raw data generation, sequencing alignments and variant calling using MPS data. However, these documents focus on requirements for human sequences rather than pathogen genomics and do not cover assembly of genomes.

Two additional standards pertain to NGS: ISO 20397, covering wet laboratory and dry laboratory sequencing methods, primarily based on human genome sequencing, including guidelines for validation of NGS data, and ISO 23418, the first standard specifically designed for sequencing of microbial pathogens, providing guidelines for sequencing and analysis of bacteria isolated in the food chain.

Accreditation is the procedure by which an authoritative body gives formal recognition that a body or person is competent to carry out specific tasks, often to ISO or ISO-equivalent standards. Accreditation is mandatory for microbiology laboratories in some countries or under some circumstances; for example, specific analyses or scenarios that affect patient management [17]. There may be a single accrediting body for a country or a range of independent third-party organizations capable of assessing the laboratory and certifying accreditation [18–23]. In Australia and the USA, accreditation is mandatory for laboratory testing services (excluding research) to receive reimbursement via government-funded health insurance programmes [21]. In Australia, an additional layer of accreditation (to higher standards) is also required for testing human samples where results may be used for clinical diagnosis or patient management [24].

## Challenges of validating pathogen genomics testing in public health

**Fig. 1.**
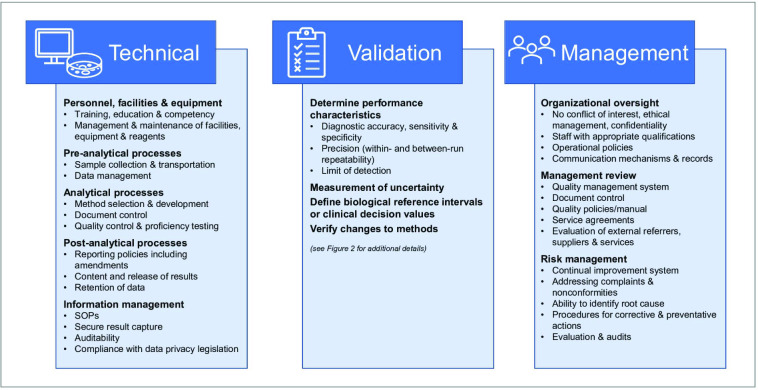
Summary of key processes for ISO accreditation. The key processes for ISO accreditation fall into three main categories: Technical, Validation and Management. Technical processes include pre-analytical, analytical and post-analytical processes, as well as management of staff and infrastructure. Validation is the process of ensuring the accuracy and suitability of new processes or equipment before introduction into the laboratory. Management processes include systems for managing quality and risk, and ensuring appropriate governance.

To gain and maintain accreditation, key management and technical processes must be established in the laboratory, including implementation of a quality management system, audit processes, validation and documentation of testing methods, training of personnel and data management (Fig. 1). These requirements can be challenging, but achievable, to implement in non-diagnostic laboratories, such as academic laboratories that wish to contribute to public health activities. In some cases, this also requires a shift in mindset; for example, privacy laws may prohibit the use of open source software tools or online data analysis programs frequently used for pathogen genomics, unless managed in a secure internal data environment. Test validations are also tied to specific software and database versions; hence reverification of tests is required after each software or database update [1].

At MDU PHL, we have been accredited for a number of pathogen genomic workflows, ranging from generation of sequence data (platform) to bioinformatic analysis of sequence data to determine antimicrobial resistance, serotype or sequence type. Validation of these test methods to provide objective evidence that the method is performing as intended is a critical component of accreditation to ISO standards. Performance characteristics around limit of detection, accuracy, sensitivity, specificity and, importantly, precision (repeatability and reproducibility) are essential to establish the utility and limitation of the method. Finally, documentation of process (standard operating procedures), establishing the training and competence of personnel, and recording and reporting of results are also important. Reporting of genomic results is particularly challenging to ensure clear unambiguous interpretations that also convey the limitations of the technology, often to non-experts.

**Fig. 2.**
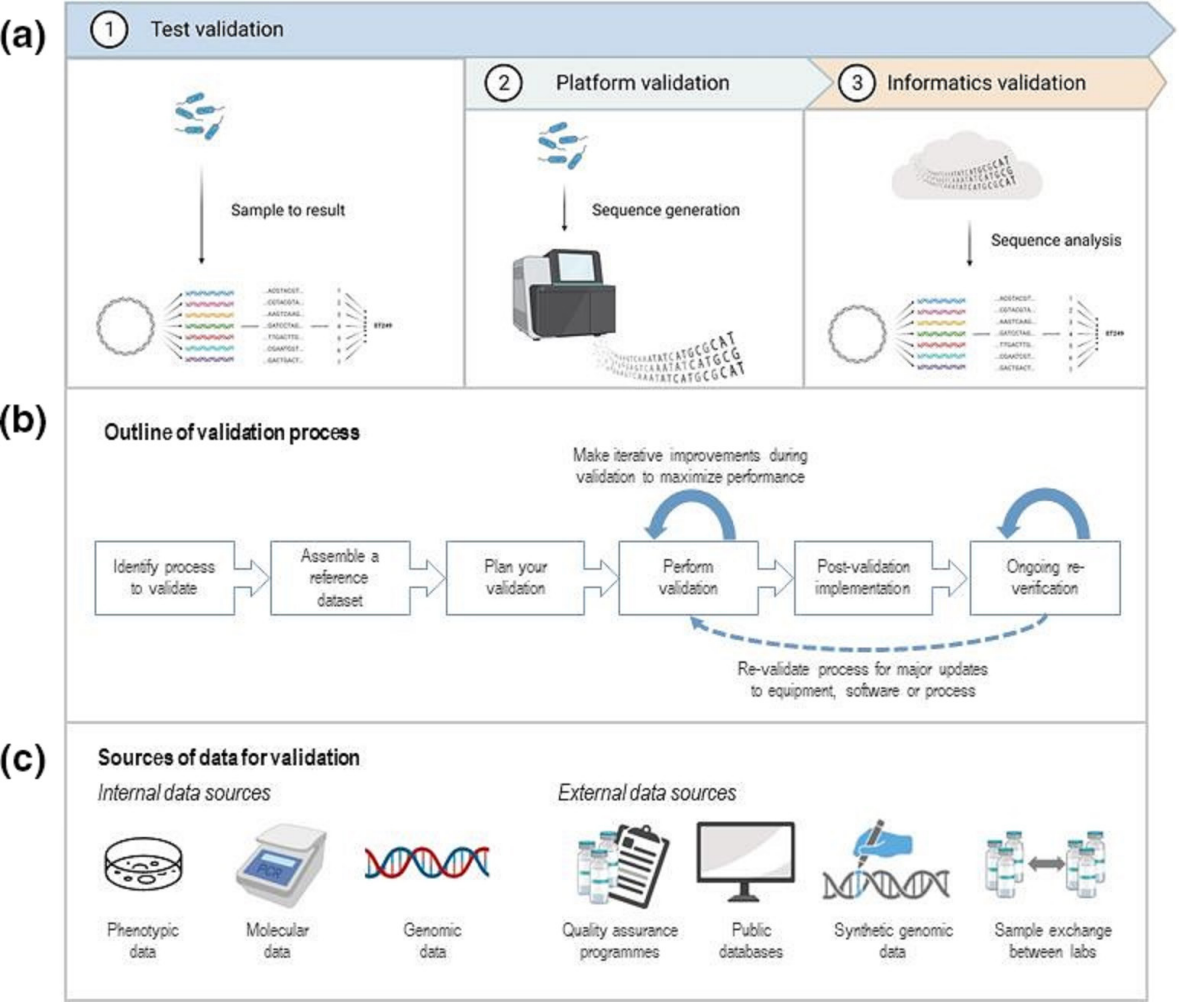
Overview of validation approaches as an essential component for ISO accreditation. (a). Test validation can be performed on multiple levels, varying from end-to-end process validation (e.g. from sample receipt to reporting of results), or can focus on individual test components, such as the sequencing platform or a bioinformatic analysis pipeline. (b). Schematic outline of the validation process, including the role of iterative improvements during validation to optimise testing, and the need for ongoing re-verification (minor changes) or re-validation (major workflow changes). (c). Examples of different data sources to assemble a reference dataset for validation, including data from your laboratory, or external data sources. Figures created with BioRender.com.

There are a number of publications around validation of whole-genome sequencing (Fig. 2), including validation of end-to-end workflows [25–28], sequencing platforms [29] and bioinformatic workflows [30–33]. Some laboratories may choose to validate wet-laboratory NGS workflows and bioinformatic analysis and reporting separately, whereas others may choose to validate the end-to-end process. One important component to consider is the use of databases of genetic targets, such as antimicrobial resistance genes or serotypes. Databases for the same targets may vary significantly in composition and annotation, hence it is important to find and use the database that is most accurate and fit for purpose for your workflows [34].

Validation of new laboratory tests usually requires comparison to a gold standard, and this is particularly challenging in pathogen genomics (Fig. 2). For many NGS applications, there are no suitable comparator assays, meaning that we need to be a bit more creative to compile a dataset covering the range of allelic targets likely to be seen for this assay – that is, to search broadly for combinations of multiple datasets, either locally or available publicly, that when combined will demonstrate the adequacy of performance of the NGS test. In particular, this may include the use of synthetic genomic data to supplement real-world data where there are no existing comparator assays [35].

Bioinformatics pipelines and tools can present particular challenges for validation. Analyses may be performed using tools or pipelines constructed in-house, downloaded from open access sources and run in-house, or run online use publicly available online tools. Importantly, attention must be paid to updates in either the tools or databases used for analysis; validation and accreditation only refer to a particular version of the tools and databases used for analysis. To maintain the validity of the test, routine (preferably semi-automated) processes for reverification of the test, usually by testing a subset of the original validation dataset, is critical. Most open-source tools and databases will notify users of any updates, but it may be more challenging to identify updates to online tools and databases, as users may specifically have to search for information on changes. Globally, we should strive for consistency in the way that these changes are communicated to users and therefore downstream impacts on users and accredited laboratories.

While publication of pathogen genomics validations are critical to inform laboratories of the performance capability of a workflow, importantly, they do not replace in-house validation. It is still a requirement of ISO standards to validate and/or verify the performance of any test (including bioinformatic tools) within your own laboratory environment and demonstrate, at minimum, equivalence to existing methods. Issues identified in validation need to be resolved and the performance metrics of sensitivity, specificity, accuracy and precision established. Acceptance and rejection criteria for valid results need to be identified and implemented within a quality systems framework. Resolution of identified issues can range from modification of the technical procedure to bioinformatic software changes. In some cases, it might not be possible to fully resolve issues, and clients may be informed when reporting results; for example (i) specifying the limitation of the assay, (ii) cautionary or explanatory notes regarding result interpretation, (iii) including clinical decision values or (iv) incorporating biological reference intervals that convey the result in the context of the limitation of the assay.

## Implications for laboratories planning to use pathogen NGS for clinical or public health purposes

Many public health laboratories are already moving towards genomics-enabled surveillance and outbreak investigation. In addition, the SARS-CoV-2 pandemic demonstrated the willingness and ability of non-diagnostic laboratories to contribute to public health through pathogen genomics, with enormous potential benefits globally. For laboratories moving into genomic sequencing for public health, it is important to approach this with a quality and accreditation framework in mind, to gain the trust of public health bodies and ensure that results are accurate and reproducible. Ideally, clinical laboratories should strive for accreditation to ISO 15189 standards, and non-clinical laboratories (i.e. those only providing sequencing services) should aim for accreditation to ISO 17025 standards; in some countries, this accreditation will be mandated. Gaining accreditation requires the implementation of the key overarching laboratory processes (Fig. 1), including quality management systems and rigorous validation processes. The implementation of metagenomics in the near future, including as a primary diagnostic tool, presents even more challenges for clinical and public health microbiology due to its complexity, and additional standards for validation and accreditation will need to be established as a matter of urgency, and communicated widely with the global community.

## Conclusions

In the next decade, pathogen genomics is likely to become a routine method for public health microbiology more broadly, and a frequently used tool in clinical microbiology. We encourage laboratories deploying the technology to approach it with a quality and accreditation framework in mind, to demonstrate the accuracy of the methods and results to all stakeholders. Critically, we encourage all pathogen genomics laboratories to openly share methods, validation datasets and learnings, to build the global knowledge base and assist other laboratories as they embark on genomics implementation.
